# Limb specific Acvr1‐knockout during embryogenesis in mice exhibits great toe malformation as seen in Fibrodysplasia Ossificans Progressiva (FOP)

**DOI:** 10.1002/dvdy.24

**Published:** 2019-03-25

**Authors:** Laura Hildebrand, Mareen Schmidt‐von Kegler, Maria Walther, Petra Seemann, Katja Stange

**Affiliations:** ^1^ Berlin‐Brandenburg Center for Regenerative Therapies (BCRT) / Charité Virchow Campus Berlin Germany; ^2^ Charité– Universitätsmedizin Berlin Berlin Germany; ^3^ Berlin Brandenburg School for Regenerative Therapies (BSRT) Berlin Germany

**Keywords:** Acvr1, cKO, FOP, skeletal malformation

## Abstract

**Purpose:**

This study analyzes *Prx1*‐specific conditional knockout of *Acvr1* aiming to elucidate the endogenous role of *Acvr1* during limb formation in early embryonic development. ACVR1 can exhibit activating and inhibiting function in BMP signaling. *ACVR1* gain‐of‐function mutations can cause the rare disease fibrodysplasia ossificans progressiva (FOP), where patients develop ectopic bone replacing soft tissue, tendons and ligaments.

**Methods:**

Whole‐mount in situ hybridization and skeletal preparations revealed that following limb‐specific conditional knockout of *Acvr1*, metacarpals and proximal phalanges were shortened and additional cartilage and bone elements were formed.

**Results:**

The analysis of a set of marker genes including ligands and receptors of BMP signaling as well as genes involved in patterning and tendon and cartilage formation, revealed temporal disturbances with distinct spatial patterns. The most striking result was that in the absence of *Acvr1* in mesoderm precursor cells, first digits were drastically malformed.

**Conclusion:**

In FOP, malformation of big toes can serve as a first soft marker in diagnostics. The surprising similarities in phenotype between the described conditional knockout of *Acvr1* and the FOP mouse model, indicates a natural inhibitory function of ACVR1. This represents a further step towards better understanding the role of Acvr1 and developing treatment options for FOP.

## Key Findings

- Limb specific conditional KO of Acvr1 leads to shortened extremities and to heterotopic cartilage and bone formation.
- Acvr1 is particularly involved in the development of the first digit.
- Phenotypic similarities between the limb specific cKO of Acvr1 and the FOP mouse model, carrying the gain of function mutation p.R206H in Acvr1, indicates a natural inhibitory function of Acvr1.

## 1 INTRODUCTION

The activin A receptor type 1 (ACVR1) is a receptor involved in bone morphogenetic protein (BMP) signaling. BMPs, together with growth and differentiation factors (GDF), transforming growth factor βs (TGFβ), activins, and Nodal belong to the TGFβ family, consisting of more than 30 members.^1^ First, BMP signaling was discovered to initiate bone formation.^2^ Only later it was shown that it is also crucial for other processes like embryonic development. Here, BMP ligands and receptors are differentially expressed, thereby forming gradients in a spatial and time‐dependent manner, which lead to the definition of the body axis, organogenesis as well as the development of the skeleton and joints.^1^ To initiate BMP signaling, ligands bind as dimers with different affinities to the BMP receptors. Upon binding, a hetero‐tetrameric complex of two type I receptors (eg, ACVR1, BMP receptor type 1A [BMPR1A] and BMPR1B) and two type II receptors (eg, BMPR2, TGFβR2, ACVR2A, and ACVR2B) is formed. Thereby, due to proximity, the glycine/serine rich (GS) domain of the type I receptors gets transphosphorylated by the constitutively active kinase domain of type II receptors. As a result, the kinase domain of the type I receptors is activated and leads to the phosphorylation of regulatory mothers against decapentaplegic homolog (R‐SMAD) 1/5/8. Together as a complex with common (co)‐SMAD4, R‐SMAD1/5/8 translocate into the nucleus, were they bind as transcription factors to the DNA and initiate activation of target genes.

Depending on the ligands, their concentration, abundant BMP type I and type II receptors as well as the repertoire of tissue‐specific transcription factors, activation of BMP signaling can lead to the initiation of a variety of downstream signals, for instance chondrogenic or osteogenic differentiation. Ligands of the aforementioned receptors are predominantly BMPs, but can also be TGFβs and Activins (eg, Activin A).^3^ In vitro studies showed that BMP2 and BMP4 prefer to form complexes with BMPR1A or BMPR1B and BMPR2, while BMP6 and BMP7 show their highest affinity to ACVR2A and ACVR2B in combination with ACVR1.^4^, ^5^, ^6^, ^7^ Activin A normally activates TGFβ signaling through its high affinity receptors ACVR2A and ACVR2B, in concert with ACVR1B and ACVR1C.

Interestingly, studies in myeloma cell lines showed that Activin A also inhibits BMP signaling of BMP6 and BMP9 through competition for their shared type II receptors.^8^ Even though the different BMP receptors and ligands can compensate for each other to a certain extent, particularly embryonic patterning is prone to malformations caused by subtle changes in BMP expression or activity. This becomes most apparent, when mutations in involved proteins disturb normal BMP signaling. One example is the rare genetic disease fibrodysplasia ossificans progressiva (FOP), which is caused by point mutations in *ACVR1*. Classical FOP is characterized by malformed great toes at birth.^9^ Later, disease progression manifests in ectopic bone formation replacing skeletal muscle, tendons and ligaments.^9^

Until now, there is no causal therapy available, for which a therapeutic effect is clearly proven. Different studies showed that FOP‐associated mutations in *Acvr1* lead to increased BMP signaling, including activation without exogenous stimulus as well as hyper‐activation in response to BMP ligands.^7^, ^10^ In addition, in FOP, ACVR1 loses inhibitory functions and instead further promotes BMP signaling.^7^, ^10^ In the past, also tissue‐specific knockout (KO) of ACVR1 has been analyzed. Here, it was shown that osteoblast‐specific KO of ACVR1 results in an increase in bone mineral density, further highlighting the inhibitory functions of ACVR1.^11^ Previously, this dual function of ACVR1 as both activator and inhibitor of BMP signaling has also been shown using the model organism *Drosophila melanogaster*.^12^, ^13^

In this study, we aimed to analyze the role of ACVR1 during limb development. As homozygous KO of *Acvr1* is lethal in mice, from stage embryonic day (E) 9.5 on,^14^ the time point at which limb outgrowth is initiated,^15^ a paired‐related homeobox gene (Prx1)‐specific KO was used. Expression of Prx1 is highly specific for mesenchymal precursor cells that are involved in preosteogenic and prechondrogenic condensation during embryogenesis.^16^

## 2 RESULTS

### 2.1 Conditional Acvr1 KO during limb development leads to great toe malformation

Mutations in *Acvr1* can lead to skeletal malformations. As *Acvr1* KO was shown to be lethal in early embryonic stage, a conditional KO (cKO) was used in this study to examine the role of Acvr1 during limb development. The limb‐specific, Cre‐dependent KO of *Acvr1* is restricted to *Prx1*‐expressing cells starting in the developmental stage E9.5.^17^ For heterozygous mice, no phenotype was observed. Homozygous mice were vital, seemed to have slightly lower body weight, groomed and mated slightly less, but still autonomously. Their extremities appeared to show normal length. It was observed that the paws of homozygous *Acvr1* cKO mice were slightly smaller and toes were bent with restricted flexibility.

**Figure 1:**
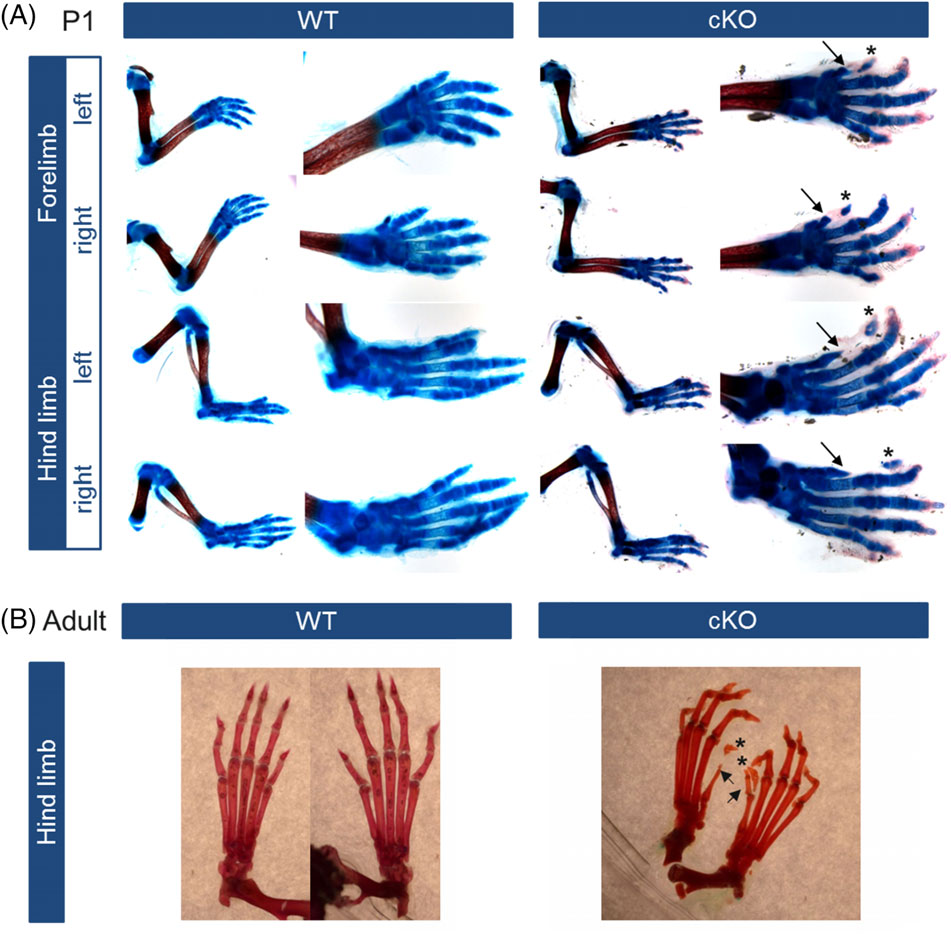
The limb‐specific conditional knockout (cKO) of *Acvr1* leads to skeletal malformations in phalanges and metacarpals. Extremities of wild‐type mice and Prx1‐Cre‐Acvr1(fl/fl) mice (cKO) were prepared and stained for cartilage (Alcian blue) and/or bone (Alizarin red) structures. **A:** After conditional *Acvr1* knockout, mice in stage P1 develop a malformation of phalangeal and metacarpal bones, whereat especially the first digit is shortened (arrow) and shows additional cartilaginous elements in the distal part (asterisk) surrounded by connective tissue. The development of stylo‐ and zeugopod of mice in stage P1 was not affected by *Acvr1* knockout. **B:** The cKO of *Acvr1* leads to a shortening of skeletal elements still found in adult mice (hind limb depicted). Phalanges of cKO mice appear bended. Again, digit 1 is most affected being drastically shortened due to missing phalanges (arrows). Additional calcified elements are found in the distal part of the digit as well (asterisks)

Skeletal preparations of newborn and adult mice elucidate disturbed development of cartilage as well as bone structures. Already at stage postnatal day (P) 1, a dramatic malformation of digit 1 was seen (Figure 1A). The metacarpal bone and the proximal phalanx were drastically shortened (Figure 1, arrows), further phalanges were not present. Additional cartilage elements were found in the far distal part of digit 1, which were surrounded by soft tissue and not connected to the proximal phalanx (asterisks). In adult mice, the first digit still showed considerable malformation (Figure 1B). The metacarpal bone mostly showed an atypical shape (Figure 1, arrows). Cartilage anlagen found in stage P1 were mineralized to form ectopic bone (asterisks).

### 2.2 Acvr1‐dependent expression of BMP ligands and receptors during limb development

**Figure 2:**
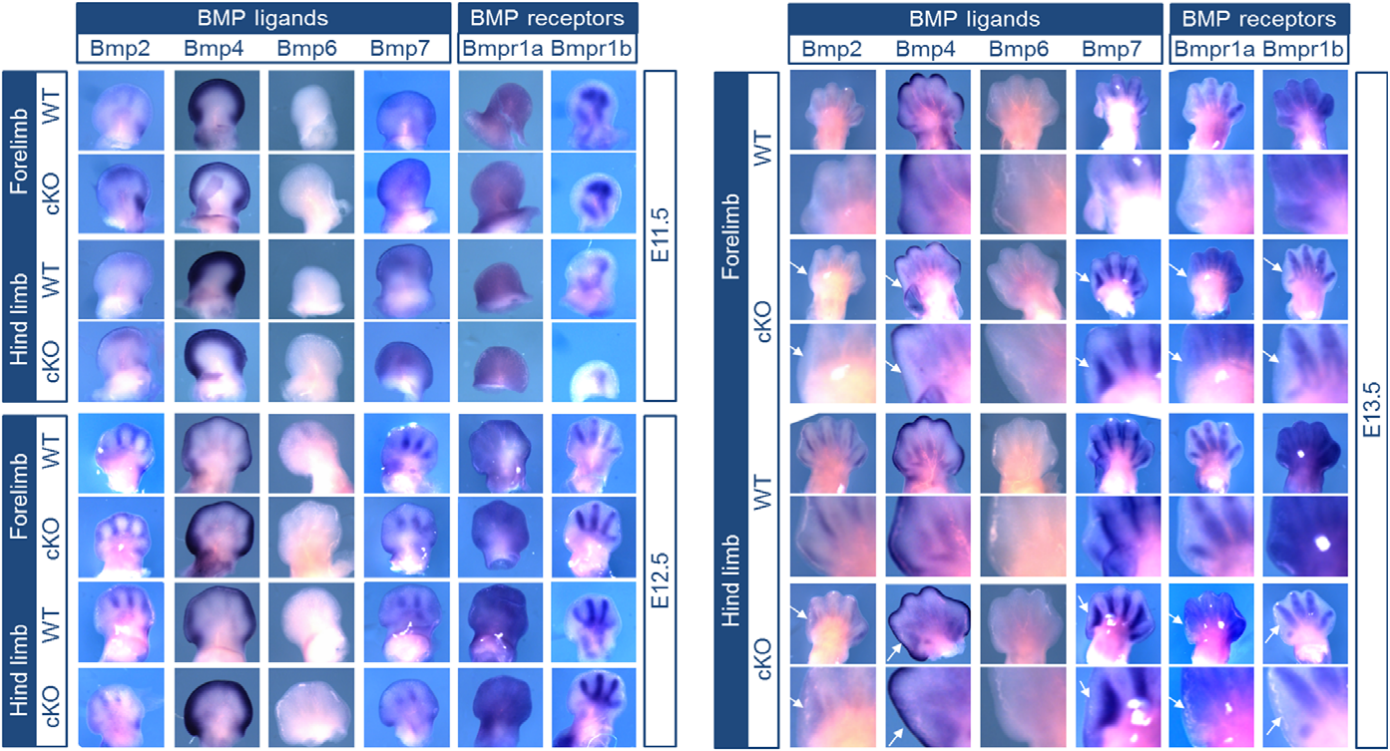
Bone morphogenetic protein (BMP) ligands and receptors are not expressed in first digits of fore‐ and hind limbs after conditional knockout (cKO) of *Acvr1*. Whole‐mount in situ hybridization (WISH) of forelimb (FL) and hind limb (HL) was performed in WT mice and Prx1‐Cre‐Acvr1(fl/fl) mice (cKO) in developmental stages E11.5, E12.5 and E13.5. Magnifications of digit 1 are provided for E13.5. Expression of BMP ligands *Bmp2*, *Bmp4*, *BMP6*, and *Bmp7* and expression of BMP type I receptors *Bmpr1a* and *Bmpr1b* were not altered by *Acvr1* cKO, except for digit 1. Interestingly, at E13.5, in digit 1 there was no expression of Bmp ligands or receptors seen in *Acvr1* cKO mice. These changes are marked with arrows

The ACVR1 receptor is part of the tightly regulated BMP signaling pathway and directly interacts with its ligands BMP6 and BMP7, but can also influence downstream signaling activity of BMP2 and BMP4 as well as their receptors BMPR1A and BMPR1B in vitro.^7^ To shed more light on this, expression patterns of these genes were qualitatively analyzed by means of whole‐mount in situ hybridization (WISH) in wild‐type (WT) and *Acvr1* cKO mice at developmental stages E11.5, E12.5, and E13.5 was performed (Figure 2).

*Bmp6* expression was not detected in the limbs at analyzed time points; therefore, no alterations, due to *Acvr1* KO, could be detected. Localization and temporal expression of *Bmp2*, *Bmp4*, and *Bmp7* as well as *Bmpr1a* and *Bmpr1b* were not influenced by *Acvr1* in digit 2‐5. In both compared mouse models, *Bmp4* was found in the apical ectodermal ridge and *Bmp7* was present in the interdigital mesenchyme (Figure 2).

Even though overall gene expression is not changed by *Acvr1* cKO, the development of the first digit of the forelimb and the hind limb appears to be abnormal. All detected members of the BMP pathway show reduced or even absent expression in this digit, predominantly seen at stage E13.5 (Figure 2, arrows). Of interest, malformations of the great toe or thumb are also frequently seen in patients carrying heterozygous *Acvr1* mutations leading to FOP.

### 2.3 Limb‐specific KO of *Acvr1* disturbs patterning, cartilage and tendon development of first digit anlage

**Figure 3:**
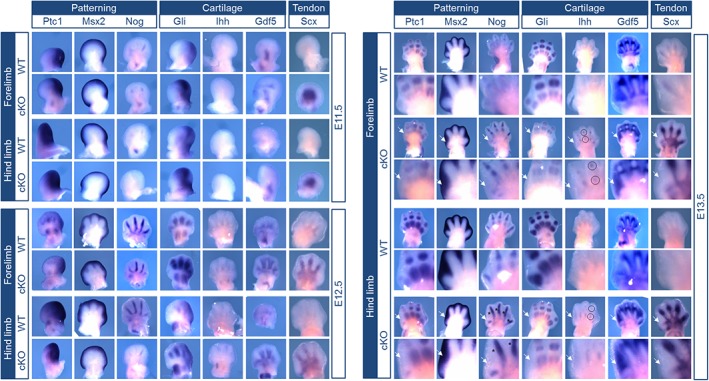
The limb‐specific knockout of *Acvr1* prevents the development of digit 1 in mouse embryos. Whole‐mount in situ hybridization (WISH) of forelimb (FL) and hind limb (HL) was performed in WT mice and Prx1‐Cre‐Acvr1(fl/fl) mice (cKO) in developmental stages E11.5, E12.5 and E13.5. Magnifications of digit 1 are provided for E13.5. Markers for patterning, cartilage and tendon development were stained. Clear changes are marked in stage E13.5 (arrow). Again, digit 1 was most affected by the cKO of *Acvr1*. The expression patterns of *Ptc1*, *Msx2*, and *Gdf5* were not altered due to *Acvr1* cKO in digits 2‐5, whereas no expression was detected in digit 1. *Nog* was only marginally expressed in digit 1 after *Acvr1* cKO and additionally, expressed in the hind limb joint interspace. Expression of the cartilage marker *Gli* was also absent in digit 1. In *Acvr1* cKO embryos, *Ihh* expression was present in the interdigital space at stage E13.5 in contrast to WT mice (exemplary highlighted for digit 4 with circle). Throughout all endpoints expression of the tendon‐specific marker *Scx* was more prominent in *Acvr1* cKO mice, however, digit 1 was excluded

Finally, markers crucial for patterning and tendon and cartilage development during embryonic limb development were selected to assess the influence of Acvr1 on their expression. WISH was performed at developmental stages E11.5, E12.5, and E13.5 (Figure 3). Gene expression in digits 2‐5 was not substantially influenced by *Acvr1* cKO. *Patched 1* (Ptc1) for instance was expressed in phalanges, *Msh Homeobox 2* (Msx2) in the interdigital mesenchyme and the developing joints were clearly marked by *Gdf5. Gli1* expression area appeared to be broader in *Acvr1* cKO mice at E11.5 and E12.5 and reduced at E13.5, pointing toward an alteration in temporal regulation, although drawing conclusions on expression intensity is limited for WISH. All analyzed genes were again not detectable in digit 1, which was the most prominent effect of the *Acvr1* cKO. Moreover, in the absence of *Acvr1*, *Indian Hedgehog* (Ihh), a key actor during induction of chondrogenesis,^18^ was still expressed at E13.5 (Figure 3, exemplarily highlighted for digit 4 with circle). In contrast to WT mice, cKO of *Acvr1* led to a continuous expression of *Noggin* (Nog) between phalanges at E13.5, especially in the hind limb (Figure 3, asterisks). *Scleraxis* (Scx), a marker for tendon development, seemed to be upregulated in *Acvr1* cKO mice.

## 3 DISCUSSION

This study analyzed the effect of a *Prx1*‐specific cKO of *Acvr1* on limb formation during early embryonic development. Already here, but also in adult mice carrying the cKO of *Acvr1*, skeletal malformations became apparent. Metacarpals as well as proximal phalanges were shortened and additional cartilage elements, later resulting in bone elements, were observed. Furthermore, a detailed analysis of a set of marker genes specific for BMP signaling, patterning as well as tendon and cartilage formation, revealed temporal and spatial disturbances, adding up to the observed phenotype.

The here‐described Prx1‐specific cKO of *Acvr1* benefits from the restriction of the depletion of *Acvr1* to mesenchymal precursor cells. Thereby, we were able to show that the expression of *Acvr1* in these precursor cells is essential for normal limb formation. These are intriguing data for ACVR1, as several reports describe that in general a compensation of one deregulated gene is possible,^19^ for example, in mice BMP5 and GDF5 can compensate for each other to a certain extent.^20^ Even though at first, consequences of the cKO of *Acvr1* seem subtle and would argue for such a compensation, as expression of *Bmp2*, *Bmp4*, *Bmp7*, *Bmpr1a*, and *Bmpr1b* is normal for digits 2‐5, a detailed look reveals that digit 1 is completely left out during early embryonic development, which became most apparent in developmental stage E13.5, surprisingly revealing a high resemblance to the malformed great toes in FOP. This is one of the most prominent and devastating diseases caused by increased BMP signaling. Here, point mutations in ACVR1 result in a gain‐of‐function, manifesting in the development of additional bone, immobile joints and heterotopic ossifications (HO) throughout life.

Until now, the described toe malformation was exclusively observed in FOP patients or after introduction of a FOP mutation into the sequence of *Acvr1* in a mouse model. For example, Chakkalakal et al. developed chimeric *Acvr1* knock‐in mice for FOP (Acvr1R206H/þ), which showed a malformation of the first digits in the hind limbs in radiographic analyses together with postnatal extra skeletal bone formation, recapitulating the human disease.^21^ Recently, it has been shown that the mutation p.R206H in ACVR1, with which the majority of FOP patients is diagnosed, leads to the loss of the inhibitory effect of ACVR1 on BMP signaling of BMP2 and BMP4 mediated by BMPR2 and BMPR1A or BMPR1B in vitro.^7^ Therefore, it is tempting to speculate that this inhibitory effect, which is no longer present, when ACVR1 is knocked out or carries a gain‐of‐function mutation, is crucial for the development of digit 1. For other digital malformations in humans, such as brachydactylies, it remains elusive, why specific digits are affected, while others develop normally.^22^

Recent publications by Hatsell et al.^23^ and Hino et al.^24^ showed that, in vitro, stimulation with Activin A induced BMP signaling in cells carrying the FOP mutation R206H or overexpressing this mutation or other less common FOP mutations, but not in WT cells. Similarly, in vivo experiments indicated that activin A induces HO, as soon as a gain‐of‐function mutation in ACVR1 is present. Olsen et al. showed that normally, Activin A inhibits SMAD1/5/8 signaling.^8^ This effect might be due to a competition for the shared type II receptor ACVR2A and ACVR2B. Therefore, this may suggest that in the Prx1‐specific *ACVR1*‐KO mouse model, depletion of ACVR1 leads to increased TGFβ signaling in that matter. Here, we concentrated on a first characterization of this model mainly with classic BMP ligands. However, as this is only one small part of a complex network of signaling pathways, it opens further research possibilities.

A lot of research focused on the search for the cell source responsible for the HO. With the help of different mouse models it could be proven that a set of cell types, including immune cells (macrophages, B and T lymphocytes), muscle precursor cells (PAX7+, MYF5+), endothelial cells (TIE2+, CADH5+), pericytes (CSPG4+, GLAST+) and vascular smooth muscle cells (SM22α+, SM‐MHC+) do not or only to a very low percentage contribute to the ectopic bone formation.^18^, ^25^, ^26^ Of interest, cells of adaptive as well as innate immune system origin also show no contribution, even though depletion of mast cells and macrophages led to a reduction of at least half of the ectopic bone in conditional‐on global knock‐in mice expressing Acvr1 R206H.^27^

Experiments in different mouse models showed that main cell source for HO are a muscle‐resident interstitial MX1+ population as well as SCX+ tendon‐derived progenitor cells.^26^, ^28^ Hereby, MX1+ cells are responsible for intramuscular, injury‐dependent endochondral HO, whereas HO of ligaments and joints without exogenous injury consists of SCX+ cells.^26^ This seems of particular significance considering that gene expression of *Scx* could also be upregulated in the here‐described cKO Acvr1 mouse model with highest abundance in the interphalangeal joints. This indicates that Scx might also be negatively regulated by ACVR1. Interestingly, animal caretakers observed that toes of cKO *Acvr1* mice seemed rather immobile and a bended positioning of toes was seen compared with WT mice, possibly caused by joint or tendon malformations. In contrast to WT mice, Scx expression seemed more prominent in cKO *Acvr1* mice during developmental stage E13.5. However, WISH provides a very useful tool for a qualitative analysis of expression patterns of genes as well as presence or absence of gene transcripts, but results only allow for qualitative analysis, as they tend to vary between experiments and no reference gene (housekeeping gene) is used. As it was shown that in vivo Scx is expressed in cells that later form tendon and ligaments,^29^ it seems that these tissues are also developing abnormal and might lead to an additional bending. Therefore, Scx seems to be a fascinating candidate for further research.

In addition to deregulation of Scx, analysis of other marker genes responsible for patterning, cartilage and tendon development revealed that here again, the anlagen for digit 1 are missing. In addition, *Nog* as well as *Ihh* were expressed at additional sites in the cKO of *Acvr1* at E13.5. In early stages (E11.5 and E12.5) *Nog* is expressed normally and joints can still be formed. Only later (E13.5) expression of *Nog* is not restricted to the phalanges, which is most apparent in the hind limb. Noggin is a well‐known inhibitor of BMP signaling,^30^ as its expression is induced by BMPs as a negative feedback mechanism and Noggin‐null mice die at birth with skeletal malformations, due to constant BMP signaling.^31^, ^32^ Ihh is critical for cartilage formation and its expression is also opposed by Noggin.^33^, ^34^ At E13.5, mice with a cKO of *Acvr1* still express *Ihh*, mostly in the interphalangeal joints. This is in contrast to WT mice, which no longer show expression of *Ihh*. This, together with an abnormal expression of *Nog* in the interphalangeal joints, might lead to subtle joint malformations of cKO *Acvr1* mice.

Later, in P1 and adult cKO *Acvr1* mice, shortened first digits as well as additional cartilage/bone elements were present. This indicates that deregulation in early stages of development, due to a lack of *Acvr1* expression, possibly cannot be compensated later. This is in accordance with studies showing that the height of the proliferative zone during endochondral ossification already determines the longitudinal growth potential of long bones.^35^ In another mouse model, which used a Col2‐specific KO of *Acvr1*, limiting its deficiency to chondrocytes, it was observed that even though all five digits were developing, their ossification was delayed.^36^ Here, ACVR1 might have a critical role in inhibiting BMP signaling to promote hypertrophy. The shortage of metacarpals as well as proximal phalanges in cKO *Acvr1* mice demonstrates that ACVR1 is critical for longitudinal growth. This is in line with Mishina et al., who reported of smaller sized *Acvr1*‐KO embryos at E7.0.^14^ The Prx1‐specific KO confirms that mesenchymal precursor cells are hereby a critical cell source.

Taken together, the newly established mouse model gives a first hint toward ACVR1 being required for correct development of digit 1. However, to support the findings presented, further studies are needed. Of special interest is the observation that cKO of *Acvr1* as well as gain‐of‐function mutations in *Acvr1*, typically present in FOP, both culminate to malformation of digit 1. In general, these are two opposing events either leading to complete loss (*Acvr1* KO) or increase (eg, FOP mutation R206H) of ACVR1 dependent BMP signaling. One explanation could be that the lack of an inhibiting function of Acvr1, due to *Acvr1* KO or an activating mutation causes the shortened digit 1. Our mouse model does not mimic the FOP phenotype but can rather help to deepen the understanding of the role of Acvr1 in vivo, which is needed to elucidate the pathomechanism underlying FOP. Regarding the genetic and molecular mechanism, the cKO of *Acvr1* is not analogous to a gain‐of‐function mutation in this receptor. Still, the here‐described mouse model and activating ACVR1 mutations show interesting phenotypic similarities by causing severe malformations in the first digit.

To assess if or to what extent the resulting phenotypes are indeed similar, further studies will be needed, also taking into account further tissue analyses including tendon and skeletal muscle. Understanding, how the blueprint for normal digit formation takes place, is essential to learn more about bone diseases like FOP. Combining the knowledge gained from Acvr1 KO studies with Acvr1 gain‐of‐function experiments will give valuable information for the role of Acvr1 and the disease mechanism of R206H, which is of importance for the development of a therapy.

## 4 EXPERIMENTAL PROCEDURES

### 4.1 Mice

Acvr1tm1Vk mice^37^ were cross‐bred with Tg(Prx1‐cre)1Cjt mice^17^ to conditionally knock out *Acvr1* in limbs ((Acvr1tm1Vk)‐(Tg[Prx1‐Cre]1Cjt)) mice, (breeding number Regional Office for Health and Social Affairs Berlin (LAGeSo): G0346/13). Exon 7 of Acvr1 is homozygously flanked by loxP sites and, therefore, can be removed by Cre recombinase, which is expressed under control of the Prx1 promoter. Paternal inheritance of Cre recombinase was necessary as maternal inheritance was lethal in combination with conditional *Acvr1* KO. Embryos were used for WISH, neonatal (P1) and adult mice (4 months old) were used for skeletal staining. All animals were genotyped after DNA isolation from tail tips (QuickExtract DNA Extraction Solution, Biozym) for *Cre* recombinase und *loxP* sites by means of polymerase chain reaction (primers: Cre fwd gagtgatgaggttcgcaaga, Cre rev ctacaccagagacggaaatc; mAcvr1 flox fwd cccccattgaaggtttagagagac, mAcvr1 flox rev ctaagagccatgacagaggttg).^38^ For control hetero‐ or homozyguous Acvr1tm1Vk mice (no Cre expression) were used.

### 4.2 Skeletal preparation

Mice were killed by fumigation with CO_2_, neonatal mice (P1) were decapitated. Afterward, hind limbs were freed from tissue as far as possible, dehydrated in 100% ethanol overnight and incubated in staining solution (0.2% Alizarin Red [Sigma‐Aldrich], 0.5% Alcian Blue [Sigma‐Aldrich], 57% ethanol, 13% acetic acid) for 3 days. Cartilage was stained in blue and mineralized tissue in red. Remaining soft tissue was removed by 1% KOH. Skeletons were incubated in increasing concentrations of glycerine and finally stored in 86% glycerine solution.

### 4.3 WISH

WISH of Prx1‐Cre‐Acvr1(fl/fl) mouse embryos was performed using digoxygenin (DIG) ‐labeled antisense probes for Bmp2, Bmp4, Bmp7, Nog, Gli1, Ptc1, Ihh,^39^ Bmp6 (provided by Andy McMahon), Gdf5,^40^ Bmpr1a,^41^ Bmpr1b,^42^ Msx2 (provided by Sigmar Stricker), Scx.^43^ Acvr1tm1Vk mouse embryos were hybridized for comparison (named wild‐type, WT). WISH was performed as previously described.^40^, ^44^ DIG‐labeled probes were detected using an anti‐DIG antibody conjugated to alkaline phosphatase (1:5000, Roche). Embryos were incubated with BM‐Purple substrate (Roche) until considerable staining was developed (8‐19 hr) and documented using Binocular MZ6 (Zeiss) and the corresponding AxioVision Software (Zeiss).

Results of this work were partly included in Stange.^45^
